# Solitary plasmacytoma: population-based analysis of survival trends and effect of various treatment modalities in the USA

**DOI:** 10.1186/s12885-016-3015-5

**Published:** 2017-01-05

**Authors:** Nishitha Thumallapally, Ahmed Meshref, Mohammed Mousa, Terenig Terjanian

**Affiliations:** 1 Department of Medicine, Staten Island University Hospital, 475 Seaview Avenue, Staten Island, NY 10305 USA; 2 Department of Medicine, Suez Canal University, Ismailia, Egypt; 3 Department of Hematology/oncology, Staten Island University Hospital, Staten Island, NY USA

## Abstract

**Background:**

Solitary plasmacytoma (SP) is a localized neoplastic plasma cell disorder with an annual incidence of less than 450 cases. Given the rarity of this disorder, it is difficult to conduct large-scale population studies. Consequently, very limited information on the disorder is available, making it difficult to estimate the incidence and survival rates. Furthermore, limited information is available on the efficacy of various treatment modalities in relation to primary tumor sites.

**Methods:**

The data for this retrospective study were drawn from the Surveillance, Epidemiology and End Results (SEER) database, which comprises 18 registries; patient demographics, treatment modalities and survival rates were obtained for those diagnosed with SP from 1998 to 2007. Various prognostic factors were analyzed via Kaplan-Meier analysis and log-rank test, with 5-year relative survival rate defined as the primary outcome of interest. Cox regression analysis was employed in the multivariate analysis.

**Results:**

The SEER search from 1998 to 2007 yielded records for 1691 SP patients. The median age at diagnosis was 63 years. The patient cohort was 62.4% male, 37.6% female, 80% Caucasian, 14.6% African American and 5.4% other races. Additionally, 57.8% had osseous plasmacytoma, and 31.9% had extraosseous involvement. Unspecified plasmacytoma was noted in 10.2% of patients. The most common treatment modalities were radiotherapy (RT) (48.8%), followed by combination surgery with RT (21.2%) and surgery alone (11.6%). Univariate analysis of prognostic factors revealed that the survival outcomes were better for younger male patients who received RT with surgery (*p* < 0.05). Additionally, patients who received neoadjuvant RT had increased survival rates compared to those receiving adjuvant RT (86% vs 73%, *p* < 0.05). Furthermore, the analyses revealed that 5-year survival rates for patients with axial plasmacytoma were superior when RT was combined with surgery (*p* < 0.05). In the multivariate analysis, age <60 years and treatment with either RT or surgery showed superior survival rates. Progression to multiple myeloma (MM) was noted in 551 patients. Age >60 years was associated with a lower 5-year survival in patients who progressed to MM compared to those who were diagnosed initially with MM (15.1 vs 16.6%). Finally, those who received RT and progressed to MM still had a higher chance of survival than those who were diagnosed with MM initially and treated with RT/surgery (21.8% vs 15.9%, *p* < 0.05).

**Conclusions:**

A review of the pertinent literature indicates that we provided the most comprehensive population-based analysis of SP to date. Moreover, our study contributes to the establishment of the optimal SP treatment modality, as RT is the favored option in frontline settings. Consensus is currently lacking regarding the benefits of combined treatment including surgery. Thus, the findings reported here elucidate the role of primary treatment modalities while also demonstrating the quantifiable benefits of combining RT with surgery in relation to different primary tumor sites. While our results are promising, they should be confirmed through further large-scale randomized studies.

**Electronic supplementary material:**

The online version of this article (doi:10.1186/s12885-016-3015-5) contains supplementary material, which is available to authorized users.

## Background

Multiple myeloma (MM) is a malignant plasma cell disorder that can result in significant organ damage. In rare cases, plasma cell proliferation occurs in the form of a solitary lesion. These presentations can be found either in bone (leading to what is called solitary bone plasmacytoma, SBP) or soft tissue (extramedullary plasmacytoma, EMP), without any evidence of systemic invasion. It is currently estimated that solitary plasmacytoma (SP) accounts for only approximately 5% of all plasma cell neoplasms that are diagnosed annually [1–3]. Due to the low incidence of this disease, empirical evidence on the clinical course and benefits of various treatment modalities is scarce. This limits the knowledge needed to determine the best course of action for individual SP patients. There is a paucity of published works on cases of SP, and most of those conducted to date have involved small cohorts, including approximately 50–250 patients [4–8]. Although these studies attempted to define the prognostic factors and establish appropriate treatment strategies, they were unsuccessful in identifying the key aspects that would delay the progression of the disease to MM. These studies were followed by large population-based studies conducted in the last decade. For example, Dores et al. based their incidence and survival rate findings on a study of 1500 patients, which is one of the largest study published to date [9]. Authors of other population-based studies evaluated either SBP or EMP in isolation, without conducting comparative analyses [10, 11].

The treatment guidelines followed by most practitioners are based on knowledge inferred either from individual case reports or from series. Currently, radiation therapy (RT) is typically offered in frontline settings, as SP is a highly radiosensitive tumor and beneficial outcomes are expected [12, 13]. However, practitioners are increasingly considering combining RT with surgery, depending on the location of the plasma cell proliferation. To date, a consensus has not been reached regarding the role of surgery in improving survival rates. Recently, Li QW et al. reported that the outcomes yielded by RT alone were superior to those achieved in SP patients who underwent surgery [14]. However, when interpreting these findings, it is important to note that their study was conducted at a single institution and included only 38 SP patients. In addition, the authors failed to assess the benefits of combined treatment (in which surgery was offered alongside RT) and to contrast those results with the outcomes of the aforementioned treatment modalities. At this time, no data exist on the survival rates of patients who received adjuvant RT relative to those of patients who were given neoadjuvant RT along with surgery.

Therefore, to gain a better understanding of the benefits of RT and surgery in patients with SP and to ascertain the relative survival rates on a larger scale, we conducted a retrospective study. The data analyzed and reported in this work were sourced from the Surveillance, Epidemiology and End Results (SEER) database, a population-based registry in the US. By including data pertaining to SP patients diagnosed in the 1998-2007 period, we have conducted by far the largest population study involving SP patients.

## Methods

The SEER program was established in 1973 by the National Cancer Institute (NCI), in accordance with the National Cancer Act of 1971 [15]. The SEER database contains information relevant to 18 distinct population groups registered in 198 US counties and currently contains data on approximately 26% of the overall US population. The value of the SEER database stems not only from the comprehensive population-based records but also from the detailed information on cancer patients. Due to its quality and extensiveness, and in particular its 98% case completeness, the SEER database serves as a benchmark against which other cancer registries are measured [16].

For the present investigation, using the International Classification of Disease for Oncology, 3rd Edition (ICD-O-3) topographical (site and morphology/primary site-labeled variable) and morphological (site and morphology *ICD-O-3* histology/behavior, malignant variable, 9731/3, 9734/3) codes, we searched the SEER database for SP cases diagnosed between 1998 and 2007. To meet the study objectives, we further assessed all identified patient records, in which we evaluated the SP according to primary tumor sites, focusing on bone (SPB, topography codes C40-41) and extramedullary sites (EMP, all topography codes excluding C40-41, C80) as the most common sites. We further classified SBP into appendicular and axial, while grouping EMP cases into those defined as upper airway, lower airway, gastrointestinal, central nervous system, skin, connective tissues and lymph nodes, based on the site of occurrence. It was noted that few patients were coded as bone marrow (c42.1) or reticulo-endothelial system (c42.2) as the primary site. However, the concurrent histology codes were 9732/3, which indicates multiple myeloma. Hence, to avoid bias, a decision was made to include codes c42.1 and c4.2 along with unknown sites (c76, c80) in one cohort called P-unspecified.

As part of this investigation, records pertaining to 1691 SP patients in the SEER database were retrospectively analyzed to obtain information on patient demographics (age, sex, and ethnicity) and treatment modalities. To facilitate subsequent analyses, patients were grouped into four age cohorts (>40, 40-49, 50-59, >60 years). The data on SP staging were limited, as “local” or “distant” were the main categories used in the database. In our work, patients with “distant” involvement were not of interest, as by definition, SP is a localized neoplastic growth. Thus, records pertaining to these patients were excluded from further analysis. The remaining cohort was analyzed using the variables “radiation,” “radiation sequence with surgery” and “Rx-summ.surg.prim.site,” allowing for additional information on treatment modalities to be sourced from SEER. Institutional review board approval was not required for this study, as SEER does not provide any patient-identifying information.

### Statistical analysis

In the present study, the aim was to assess the relative survival of patients who had undergone different treatment modalities. This primary outcome measure was presented as the ratio of the overall survival and the expected survival (within a general population cohort) [17]. Once this information was obtained, survival curves were generated by applying the Kaplan-Meier method and were compared using Mantel-Cox log-rank test. One-way analysis of variance (ANOVA) assisted in the comparison of multiple groups. A multivariate analysis was employed to determine the independent prognostic factors using Cox proportional hazards model. In addition, the disease incidence rates were age-adjusted for the aforementioned four groups (<40, 40-49, 50-59, >60 years) and were normalized using the 2000 Standard US population. Statistical analyses were conducted using SEER*Stat Limited Use software provided by the National Cancer Institute, Bethesda, Maryland. In addition, GraphPad Prism (GraphPad Software, Inc, La Jolla, California, USA) and IBM SPSS 20 were employed to analyze and present the data, in which *p* < .05 was considered statistically significant.

### Results

Demographic characteristics of the patients included in this retrospective study are summarized in Tables 1 and 2. The median age at diagnosis was 60.38 ± 14.22 years, which is 10 years lower than that of the age at diagnosis of MM. In terms of gender, our patient cohort was not evenly distributed, as 62% of the patients were male. The male to female ratio of our cohort was 1.7:1. The cohort was predominantly Caucasian (80%) followed by African American (14%) and other races (5.4%). Bone was the most common site of involvement (57.78%). Additionally, 831 (49%) and 146 (8%) patients had axial and appendicular skeletal involvement, respectively, while plasma cell proliferation in soft tissues was noted in 540 patients (32%). In patients with EMP, the most commonly encountered site was the upper airway tract (12%).

Table 3 presents the treatment modalities used in different primary sites and shows that 825 patients (49%) received radiotherapy and 197 (12%) underwent surgery, while 359 patients (21%) required both RT and surgery. In most cases, SP was treated by RT alone, with fewer patients receiving a combined treatment or surgery only. We further analyzed the data for patients receiving both treatment modalities, aiming to establish any differences in relative survival between those who were given RT in neoadjuvant vs adjuvant settings.

The overall incidence of plasmacytoma in 2007 was 0.3745/100,000, which indicated a slight increase relative to the incidence in 2005, which was reported to be 0.3462/100,000. The incidence rate ratio of plasmacytoma for African Americans to Caucasians was calculated, yielding a ratio of approximately 1.25. The prognostic factors of 5-year relative survival are summarized in Table 4. With a median follow up of 9.7 years, the overall median survival of the entire cohort was 8.12 years. As seen from the results, the highest survival rates were noted for the younger age group (<40 years), while patients aged 60 years or older had the most unfavorable outcomes (87.45% vs 45%, *p* < .05). Moreover, males had a higher chance of survival than females (63.7% vs 52.9%, *p* < .05), and more favorable outcomes were recorded for Caucasians and patients of other races relative to the African American cohort. However, these differences were not statistically significant (59.1% vs 57.6%, *p* = .083) (Additional file 1: Figure S1, S2, S3).

**Table 1:** Patient demographic characteristics

Base line characteristics		Frequency	Percent
Age	>40	133	7.9%
	40-49	231	13.7%
	50-59	379	22.4%
	≥60	948	56.1%
Sex	Male	1056	62.4%
	Female	635	37.6%
Race	Caucasian	1353	80%
	African American	247	14.6%
	Others	91	5.4%
Exposure To Radiotherapy	Without Radiation	507	30%
	With Radiation	1184	70%
Radiation Sequence	Adjuvant	342	20.2%
	Neoadjuvant	18	1.1%
	Neoadjuvant and adjuvant	5	0.3%
Surgical Intervention	No	1135	67.1%
	Yes	556	32.9%

**Table 2:** Distribution of the study population according to the primary tumor site

Site	Number (n)	Percentage (%)
Bone (SBP)	977	57.78%
Axial skeleton (c41)	831	49.14%
Appendicular skeleton (c40)	146	8.63%
Extramedullary plasmacytoma(EMP)	540	31.93%
Upper airway tract (Sino-nasal mouth and pharynx) (c00-14, c33-39)	211	12.48%
Lower airway tract (c32-39)	54	3.19%
Gastrointestinal Tract (c15- 26)	49	2.9%
Soft tissue and connective tissue (c49)	77	4.55%
Central nervous system (c69-72)	49	3.19%
Lymph nodes (c27)	25	1.48%
Skin (c44)	20	1.18%
All other sites (c48,50,53,56,67,68,73,74)	55	3.25%
Plasmacytoma Unspecified (P-unspecified) [Hematopoietic and reticulo-endothelial (c42), unknown (c76,80)]	174	10.29%
Total	1691	100%

**Table 3:** Radiation and surgical intervention according to primary tumor site

Site	Both (%)	Radiation only (%)	Surgery only (%)	Neither rad. nor surgery (%)	Total (%)
Bone (SBP)					
Axial skeleton (c41)	177 (21.3%)	481 (57.88%)	58 (6.98%)	115 (13.84%)	831 (49.14%)
Appendicular skeleton (c40)	30 (20.55%)	78 (53.42%)	15 (10.27%)	23 (15.75%)	146 (8.63%)
Extramedullary plasmacytoma(EMP)					
Upper airway tract [Sino-nasal mouth and pharynx (c00-14, c33-39)]	86 (40.76%)	77 (36.49%)	30 (14.22%)	18 (8.53%)	211 (12.48%)
Lower airway tract (c32-39)	5 (9.26%)	19 (35.19%)	9 (16.67%)	21 (38.89%)	54 (3.19%)
Gastrointestinal Tract (c15- 26)	2 (4.08%)	11 (22.45%)	24 (48.98%)	12 (24.49%)	49 (2.9%)
Soft tissue and connective tissue (c49)	10 (12.99%)	33 (43.86%)	10 (12.99%)	24 (31.17%)	77 (4.55%)
Central nervous system (c69-72)	17 (34.69%)	15 (30.61%)	16 (32.65%)	1 (2.04%)	49 (3.19%)
Lymph nodes (c27)	10 (40%)	5 (20%)	5 (20%)	5 (20%)	25 (1.48%)
Skin (c44)	4 (20%)	6 (30%)	8 (40%)	2 (10%)	20 (1.18%)
All other sites (c48,50,53,56,67,68,73,74)	7 (12.73%)	14 (25.45%)	20 (36.36%)	14 (25.45%)	55 (3.25%)
Plasmacytoma Unspecified (P-unspecified)	11 (6.67%)	86(49.41%)	2 (1.21%)	75 (43.11%)	174 (10.28%)
Total	359 (21.23%)	825 (48.79%)	197 (11.65%)	310 (18.33%)	1691 (100%)

**Fig. 1:**
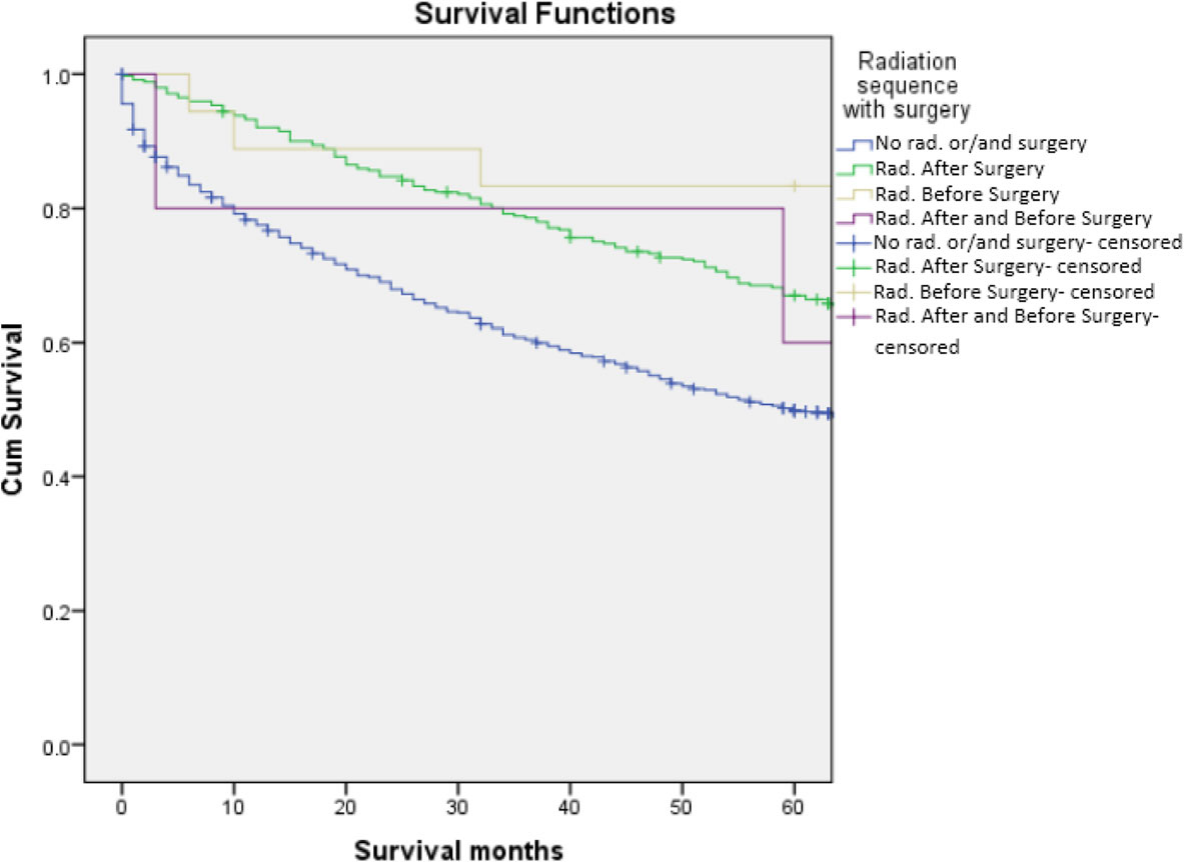
Kaplan-Meier survival curve comparing patients who received RT vs. no RT

**Fig. 2:**
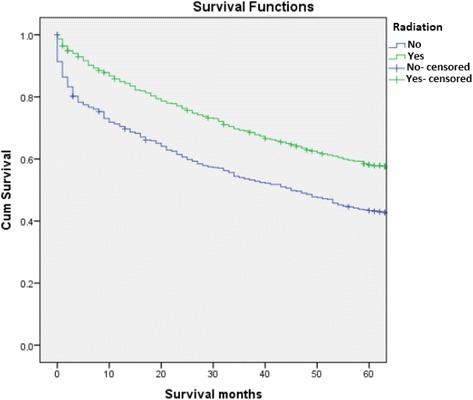
Kaplan-Meier survival curve comparing patients who received surgery vs. those that did not

**Fig. 3:**
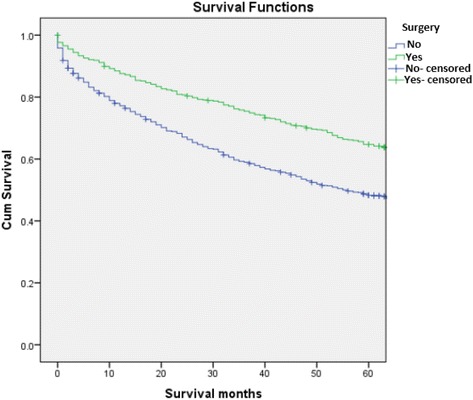
Kaplan-Meier survival curve comparing sequence of RT in patients who received RT and surgery

Analyses further revealed that the survival rates of patients treated with RT were significantly higher than those of patients who did not receive RT (64.4% vs 48.6%, *p* < .05). Moreover, patients who received neoadjuvant RT had a greater chance of 5-year relative survival than those who received adjuvant RT (86% vs 73%, *p* < .05). A significant difference in survival outcomes was also observed in patients who underwent surgery when compared to their counterparts who did not (69.7% vs 57.4%), *P* <0.05) (Figs. 1, 2 and 3). After analyzing the 5-year relative survival of different treatment modalities in relation to primary site, we noted that patients with axial skeletal involvement treated with a combination of RT and surgery had a higher survival rate (70.5%) than those who received only RT (61.5%) or surgery alone (46.4%) (*p* < .05) (Table 5). Patients with upper and lower airway tract involvement had a higher survival with surgery alone when compared to combination therapy and RT (96.7%, 100% *p* <0.05 and *p* < 0.01, respectively). Patients with involvement of the appendicular skeleton (63.6%), central nervous system (92.6%) and other sites (excluding GI, skin and connective tissues, lymph nodes) (64.4%) had an increased survival rate when treated with radiation alone (*p* = 0.024, *p* < .05, *p* 0.004, respectively). In contrast, patients diagnosed with soft tissue EMP and lymph node EMP attained better survival outcomes when given combination treatment. However, it should be noted that the differences were not statistically significant (*p* = 0.69, *p* = 0.83, respectively) (Table 5).

**Table 4:** Variable analyses of prognostic factors

Characteristics		Over all 5 years relative survival		Over all mean survival		*P* value
		%	95% CI	Month	95% CI	
Age	>40	87.4	80 – 92.2	149.87	139.88 – 159.86	<0.05
	40-49	78.6	72.4 – 83.6	130.16	120.96 – 139.35	
	50-59	73.2	67.9 – 77.7	119.70	112.56 – 126.84	
	≥60	45	41.1 – 48.7	62.56	58.25 – 66.87	
Sex	Male	63.7	60.2 – 66.9	97.03	92.35 – 101.7	<0.05
	Female	52.9	48.3 - 57.2	80.86	75.34 – 86.38	
Race	White	59.1	56 – 62.1	90.73	86.65 – 94.81	0.083
	Black	57.6	50.3 – 61.2	84.52	75.78 – 93.27	
	Others	72.9	61.1 – 81.6	111.97	96.59 – 127.36	
Exposure To Radiotherapy	Without Radiation	48.6	43.6 – 53.4	74.59	67.93 – 81.26	<0.05
	With Radiation	64.4	61.1 – 67.5	98.9	94.59 – 103.21	
Radiation Sequence	Adjuvant	72.5	66.5 – 77.6	115.12	107.43 – 122.82	<0.05
	Neoadjuvant	86.2	55 – 96.4	132.33	103.73 – 160.92	
	Neoadjuvant and adjuvant	62.8	11.5 – 90.5	72.4	38.95 – 105.85	
Surgical Intervention	No	54.7	51.3 – 57.9	81.58	77.2 – 85.97	<0.05
	Yes	69.7	65.1 – 73.8	111.37	105.11 – 117.63	

In the multivariate Cox regression analysis of the prognostic factors, we found that age < 60 years (HR 0.38, 95% CI 0.330-0.445, *p* < 0.001) and treatment with either RT (HR 0.597, *p* < 0.001) or surgery (HR 0.764 *p* < 0.001) were all independent predictors of higher overall survival. Combination therapy did not appear to be significant in the multivariate analysis (HR 1.226, 95% CI 0.966-1.552, *p* = 0.094) (Table 6).

To evaluate plasmacytoma as a localized disease with cases progressing to myeloma, information on the survival and cause of death was obtained regarding all cases of multiple myeloma during the years 1998–2007 (17,405 cases). It was noted that 553 patients (32.7%) had progressed to MM during the median follow-up of 9.7 years. Of these patients, 72% were > 60 years, 56.6% were male, and 81.2% were Caucasian (Additional file 2: Table S1). Additionally, 70% of patients who progressed to myeloma had received RT and 26.4% had received surgical intervention. We found that there was a significant difference in 5-year survival between patients >60 years who were initially diagnosed with MM compared to those initially diagnosed with plasmacytoma and who subsequently progressed to MM (16.6% vs 15.1%, *p* < 0.031). An increased 5-year survival was noted in males, Caucasians and those who received RT among patients initially labelled as SP who later progressed to MM when compared to those with an initial diagnosis of MM (22.2% vs 18%, 18.4% vs 17.5%, 21.8% vs 15.9% and *p* < 0.09, *p* < 0.018, *p* < 0.05, respectively) (Table 7).

## Discussion

MM is a systemic plasma cell disorder accounting for 1.5% of all cancers and contributing up to 13% of all hematologic malignancies worldwide. SP, while rare, is categorized as an independent subset of plasma cell disorders manifested through localized proliferation of neoplastic monoclonal cells. Despite some notable similarities between SP and MM in terms of their cytological and immunophenotypic characteristics, these tumors are differentiated by their site predilection and mortality rates. An extensive body of literature on MM exists; thus, its pathogenesis, epidemiology, and response to various treatments has been comprehensively investigated. In contrast to MM, SP has been the subject of limited research to date. Consequently, and due to its low incidence, current SP treatment guidelines are based on the findings of a limited number of retrospective studies involving small samples. Empirical evidence confirms the benefit of RT in SP patients. However, consensus regarding the effectiveness of surgery is presently lacking. A search of the SEER database revealed that more than 1600 patients were diagnosed with SP from 1998 to 2007 in the present investigation. In this study, which is, to the best of our knowledge, the largest population study reported to date, we aimed to assess the survival patterns according to age, gender, site of involvement and treatment modality as well as to examine the disease progression to myeloma.

**Table 5:** Five year relative survival pertaining to different treatment modalities according to the primary tumor site

Site	Relative survival				*p* Value
	Both (%)	Radiation only (%)	Surgery only (%)	Neither rad. nor surgery (%)	
Bone (SBP)					
Axial skeleton (c41)	70.50%	61.20%	46.4%	36.90%	< .05
Appendicular skeleton (c40)	54.00%	63.6%	37.8%	32.5%	.024
Extramedullary plasmacytoma(EMP)					
Upper respiratory tract[sinonasal mouth and pharynx (c00-14, c33-39)]	93.30%	73.8%	96.7%	45.1%	< .05
Lower respiratory tract (c32-39)	95.0%	42.3%	100%	33.5%	.001
Gastrointestinal Tract (c15-26)	0.00%	33.7%	71.6%	28.0%	.057
Soft tissue and connective tissue (c49)	61.3%	50.9%	43.2%	38.1%	.346
Central nervous system (c69-72)	67.8%	92.6%	52.9%	0.00%	< .05
Lymph nodes (c27)	74.8%	83.9%	63.0%	100.0%	.836
Skin (c44)	89.5%	90.6%	91.0%	100.0%	.692
All other EMP sites(c48,50,53,56,67,68,73,74)	31.1%	64.4%	62.3%	28.6%	.004

Given the low incidence of SP, the diagnostic criteria have been difficult to establish and have been modified throughout the years. The current guidelines provided by the International Myeloma Working Group (IMWG 2013) define SP as biopsy-confirmed plasma cell proliferation in the bone or soft tissue in the presence of normal bone marrow and a normal skeletal survey (except for the primary solitary lesion) [18]. Guidelines also recommend using the term SP with minimal bone marrow involvement when there is < 10% plasma cell proliferation in bone marrow in the presence of a confirmed solitary lesion elsewhere. These guidelines are highly helpful, as the opinions of specialists regarding the threshold for diagnosing SP with marrow involvement previously differed, ranging from 5% to 10% of plasma cell proliferation in marrow [1, 2]. Although > 10% plasma cell proliferation in bone marrow has always been indicative of MM, there had been no mention of the minimum percentage required for the diagnosis in prior IMWG guidelines (2003). These differences in opinion are noteworthy, as our study sample included patients who were diagnosed prior to the introduction of the 2013 IMWG guidelines, which resulted in a misclassification of some cases in which bone marrow was coded as the primary site. In addition, there is also the potential for misdiagnosed MM. Hence, to avoid propagating these errors in our findings, patients who were coded as bone marrow (c42.1), reticulo-endothelial system (c42.2) and unknown (c 76, c 80) were combined into one cohort, called P-Unspecified. In their study, Dores et al. introduced a similar cohort, in which all cases coded to bone marrow and unknown sites were also grouped under a P-unspecified category [9].

**Table 6:** Cox proportional hazards model for prognostic factors

Variables	Hazard ratio	95% CI		*P* value
		Lower	Upper	
Age <60 years	0.380	0.330	0.440	>0.001
radiation sequence with surgery	1.226	.966	1.557	.094
Radiation	0.597	0.522	0.684	>0.001
Surgery	0.746	0.693	0.802	>0.001

Our analyses revealed that SBP primarily involves the axial skeleton. This finding supports previously reported results, which suggested that SBP is frequently observed in marrow-containing bones, specifically vertebrae [19]. In addition, and in accordance with the findings reported by Patel et al., we found when analyzing our sample that the upper airway tract, including the sinuses, which have extensive lymphatic supply, was the most commonly involved EMP site [20].

Prior population studies have revealed a male predominance in the SP cohort. Our findings concur with this conclusion, with a male-to-female ratio of 1.6:1 [4, 20]. In existing studies focusing primarily on SBP, the male-to-female ratio was in the range of 1.5–2.4 [5, 6]. However, among EMP patients, a 3:1 male predominance was previously observed in sites involving upper airway tract [5, 20]. We also noted a gender bias in the survival rates, in which males achieved significantly better outcomes than females (64.1% vs 53.8%, *p* < 0.05). Previous research, however, did not offer conclusive findings in this respect, as men with SP showed an increased chance of survival in some studies, while no gender-related differences were reported in others [5, 9].

The majority of the patients included in our retrospective study were Caucasian, followed by African American and other races. Our results concur with those reported by Dores et al. and Jawad et al., who noted that the SP incidence rates were notably higher among African Americans than among Caucasians (IR 0.6 vs 0.4) [9, 10]. Authors of other studies focusing on MM also reported the same racial distribution [21, 22]. This observation might point to differences in genetic susceptibility between different races. However, when analyzing the SEER sample, no racially biased survival advantages could be found. When interpreting this finding, it is important to acknowledge the limited data on survival differences in different racial groups. Thus, further studies are warranted to confirm our findings.

**Table 7:** Multivariate analysis of 5 year survival of the patients diagnosed with plasmacytoma who progressed to myeloma & those with initial diagnosis of Myeloma

Characteristics		Plasmacytoma		Myeloma		*P* value
		%	95% CI	%	95% CI	
Age	>40	54.3%	24.9 – 76.5	31.2%	24.8 – 37.7	0.06
	40-49	34%	19.6 – 48.9	23%	20.5 – 25.6	0.12
	50-59	24.6%	16.5 – 33.5	23.5%	21.9 – 25.1	0.27
	≥60	15.1%	11.4 – 19.3	16.6%	14.8 – 18.4	0.031
Sex	Male	22.2%	17.3 – 27.4	18%	17.2 – 18.9	0.009
	Female	15.9%	11.2 – 21.2	16.9%	16 – 17.8	0.102
Race	White	18.4%	14.6 – 22.4	17.5%	16.8 – 18.3	0.018
	Black	23%	14.1 – 33.3	17.6%	16.2 – 19.2	0.085
	Others	27.1%	9.7 – 48.2	15.4%	14 – 19	0.104
Exposure To Radiotherapy	Without Radiation	13.6%	8.5 – 19.9	18%	17.3 – 18.8	0.062
	With Radiation	21.8%	17.5 – 26.5	15.9%	14.7 – 17.1	<0.05
Radiation Sequence	Adjuvant	22.4%	14.1 – 31.9	18.8%	12.4 – 26.3	0.096
	Neoadjuvant	100%	N/a	0%	N/a	N/a
	Neoadjuvant and adjuvant	0%	N/a	0%	N/a	N/a
Surgical Intervention	No	18.5%	14.5 – 22.8	17.5%	16.8 – 18.1	0.080
	Yes	22.1%	15.4 – 29.7	19.6%	12.7 – 27.7	0.119

*N*/*a* Not applicable

SP affects primarily those above 40, and its incidence increases with age. In our sample, the median age at SP diagnosis was 60 years, with a standard deviation of 14 years, which is consistent with previous reports. Our analyses further revealed that the survival rates were inversely related to age. Specifically, the highest and lowest chances of survival were observed for the <40 and >60 age cohorts, respectively. In accordance with the findings of previous retrospective studies reported by Knobel et al. and Jawad et al., we found that older age (>60 years) was a prognostic factor of poor outcomes in both the univariate and multivariate analyses in terms of overall survival and progression to MM [5, 10]. There could be various reasons for this observation. In patients who progress to MM, it is possible that older patients are treated with less aggressive therapies and are less involved in clinical trials compared to younger patients [5]. That being said, it remains a challenge to identify an exact reason why patients > 60 years develop MM more frequently than younger patients.

As SP is a highly radiosensitive tumor, most patients undergo RT as the first line of treatment, resulting in an excellent local control rate. In several retrospective studies, RT was found to be more effective than both surgery and chemotherapy as the sole treatment modality [5, 12, 13, 23]. In our study, an improved overall survival was noted in patients who received RT in both the univariate and multivariate analyses (Table 4). This was not in accordance with results reported by Jawad et al. and Knobel et al. [5, 10]. However, a recent retrospective study by Finsinger et al., who evaluated 53 patients, found there was a significant difference in progression-free survival (PFS) between patients who received RT and those who received other treatment modalities [24]. Despite the excellent local control, in our study, at least 70% of patients who received RT progressed to myeloma. Our study series also revealed that despite progressing to myeloma, patients who received RT still had a higher chance of survival than those who were diagnosed with MM initially and exposed to RT. To date, there are no set guidelines on the dose of RT and its effects on local control and progression of the disease. Some authors suggest using doses of 40-50 Gy for lesions smaller than 5 cm and >50 Gy for lesions >5 cm [5, 12, 23]. Tsang et al. reported that there was no evidence of improvement with RT doses > 30-35 Gy in terms of achieving local control [25]. We could not study the correlation between RT dose and local failure or progression to MM in our patients due to a lack of information in the SEER database.

Surgery for SBP patients is suggested only when they present with limb-threatening situations, such as cord compression or impending fracture. However, some authors believe that high-dose RT combined with surgery achieves a better progression-free survival relative to RT alone in SBP patients [26]. Bataille et al. and Frassica et al. reported higher rates of local failure in patients with vertebral lesions who were treated with RT only [8, 27]. These assertions were confirmed in our study, as the analyses revealed greater survival rates in patients with axial skeletal involvement treated with RT along with surgery when compared to those who underwent RT only or surgery alone.

Experts have yet to reach a consensus regarding the optimal treatment modality for EMP. Current guidelines stipulate that head and neck EMP be treated with RT only, as surgical resection of these tumors can be highly invasive [23]. However, when EMP is located in other areas, surgical resection is recommended and should be followed by RT only if the surgical margins are inadequate [28]. Sasaki et al. noted that patients who underwent surgery combined with adjuvant radiotherapy had superior survival outcomes than those receiving RT or surgery as stand-alone treatment [29]. In contrast, Alexiou et al. reported evidence indicating that offering surgery alone to EMP patients was the most beneficial treatment mode when negative surgical margins could be achieved [7]. Our analysis showed that patients with airway tract involvement (upper and lower) had overall improved survival outcomes with surgery as opposed to combination therapy with RT and surgery. The univariate and multivariate analyses in this study showed a significant survival advantage associated with surgery alone for SP. This result reflects the fact that we evaluated both SBP and EMP together as one cohort. It is unclear if the same holds true when SBP and EMP are studied individually. Furthermore, when analyzing progression to myeloma, surgical intervention did not improve survival rates. Considering the findings reported here together with the aforementioned studies, it can be asserted that for EMP patients, the best treatment modality remains elusive. Thus, further randomized controlled studies must be conducted to gain more information on this disease and the treatment outcomes.

We also found that among the SP patients who required both surgery and RT, those who received RT in the neoadjuvant setting had higher survival rates than those who were given RT in the adjuvant setting. This comparison, however, lacked sufficient statistical power, as only a few patients had received neoadjuvant RT. This may suggest the presence of a large tumor, requiring shrinkage prior to surgery, thus increasing the likelihood of efficacy of neoadjuvant chemotherapy. Chemotherapy, although not a standard of care, has been suggested by some authors. There has been only one relevant prospective trial thus far, and it evaluated the benefit of chemo radiotherapy in 53 patients. Although the study included a limited number of patients, it concluded that combination therapy increased survival duration as well as remission [30]. Kumar et al. presented in a series of 25 SBP patients that angiogenesis was highly associated with disease progression to myeloma [31]. Therefore, targeting angiogenesis with agents such as thalidomide represents a new therapeutic approach for patients with SBP.

Although, high local control rates are achieved with RT and/or surgery, progression to myeloma is still frequent. In our study, the cause of death in 33% of patients diagnosed with SP was progression to MM. Soutar et al. reported that SBP progressed to MM in >75% of cases [23]. As our cohort included both SBP and EMP, this number appears to be lower. There is no clear evidence on the factors predicting the progression of SP into myeloma. This lack of definitive evidence is attributed to the low incidence of the disease and the lack of randomized controlled studies. A few researchers consider age, size of the tumor, and persistence of M protein after treatment as factors indicating a worse prognosis. In our study, we found only age >60 to be a significant prognostic factor of progression to myeloma. As no information was available in the database regarding prior medical history, laboratory work-up and radiologic evaluation, our study could not analyze the prognostic significance of tumor size, number of lesions and persistence of M protein after treatment and accuracy of MRI.

The strengths of our study stem from the analysis of reliable population-based data, ensuring that our findings can be generalized to the US population. In addition to providing representative findings, we studied the efficacy of primary treatment modalities in relation to different sites, which can provide guidelines for clinical practice. However, it is also important to note the study limitations, one of which was the inability to review the pathology of cases and to determine the extent of bone marrow or other organ involvement (anemia, renal insufficiency). As this was a retrospective study, the accuracy of the data was beyond our control, and there is also the possibility that in some cases, MM was misclassified as plasmacytoma. However, as our analysis period ranged from 1998 to 2007, this choice reduced the likelihood of misclassification, as sensitive diagnostic testing with MRI and monoclonal paraproteins was widely available at the time. Nonetheless, to avoid potential biases, we combined the records of patients with tumors coded to bone marrow and unspecified sites into a single cohort, denoted P-Unspecified, and assessed them separately from other groups. Another noteworthy limitation stems from the absence of information on the chemotherapy regimens used in the adjuvant setting. This omission prevented us from determining the survival benefit derived from chemotherapy, if any. Lastly, we could not study the effects of other prognostic factors such as tumor size and presence of M protein after treatment in progression of SP to MM.

## Conclusion

The role of surgery in SP treatment remains controversial due to the incongruent findings reported by a limited number of studies. Based on our large population study, we conclude that therapy of combined RT with surgery is beneficial in axial plasmacytoma. Although increased survival rates were observed for patients treated with RT or surgery, disease progression to myeloma was still evident. Large prospective trials are needed to establish sound evidence on the prognostic factors associated with disease progression. Furthermore, the efficacy of the use of adjuvant chemotherapy should be established in phase II or III trials. Although our findings are highly informative, they should be confirmed with further randomized trials.

## Additional file

Additional file 1: Kaplan-Meier survival curve for patients with SP in various age groups. (DOCX 140 kb)
Additional file 2: Characteristics comparison between plasmacytoma & myeloma which progressed to myeloma. (DOCX 17 kb)

## Abbreviations

EMP: Extra medullary plasmacytoma
IMWG: International Myeloma Working Group
MM: Multiple myeloma
N/a: Not applicable
RT: Radiotherapy
SBP: Solitary bone plasmacytoma
SEER: Surveillance Epidemiology End Results
SP: Solitary plasmacytoma
