# Erratum to: Solitary plasmacytoma: population-based analysis of survival trends and effect of various treatment modalities in the USA

**DOI:** 10.1186/s12885-017-3427-x

**Published:** 2017-06-23

**Authors:** Nishitha Thumallapally, Ahmed Meshref, Mohammed Mousa, Terenig Terjanian

**Affiliations:** 1 Department of Medicine, Staten Island University Hospital, 475 Seaview Avenue, Staten Island, NY 10305 USA; 2 Department of Medicine, Suez Canal University, Ismailia, Egypt; 3 Department of Hematology/oncology, Staten Island University Hospital, Staten Island, NY USA

## Erratum

After publication of the original article [1] the authors found the following errors had occurred:

1. Figure titles were incorrect:

- Figure 1 should be titled: Kaplan-Meier survival curve comparing sequence of RT in patients who received RT and surgery (Fig. 1)
- Figure 2 should be titled: Kaplan-Meier survival curve comparing patients who received RT vs. no RT (Fig. 2)
- Figure 3 should be titled: Kaplan-Meier survival curve comparing patients who received surgery vs. those that did not (Fig. 3)

2. In Table 6, all *P* values should be <0.001 instead of >0.001 (Table 6)

**Fig. 1:**
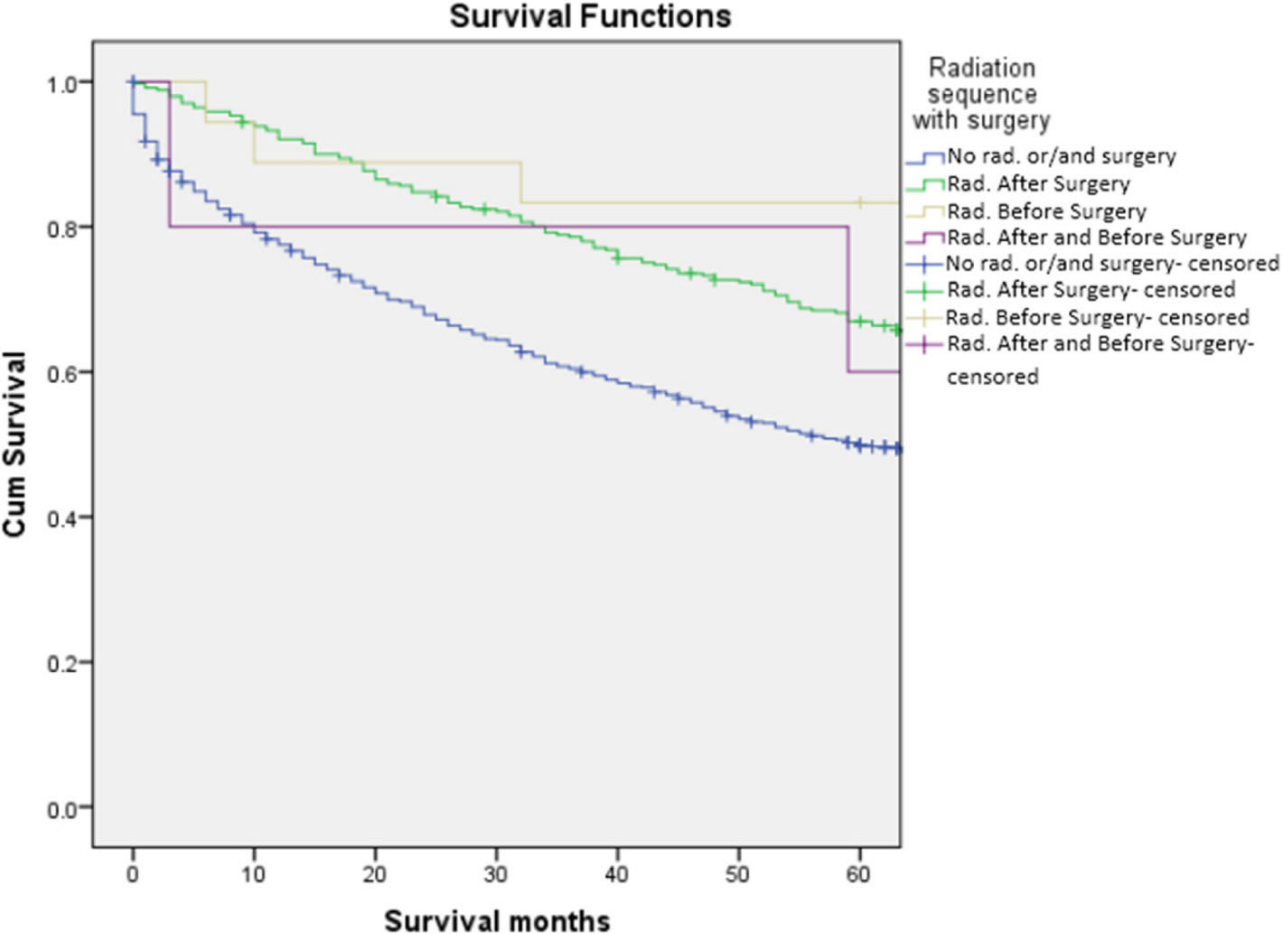
Kaplan-Meier survival curve comparing sequence of RT in patients who received RT and surgery

**Fig. 2:**
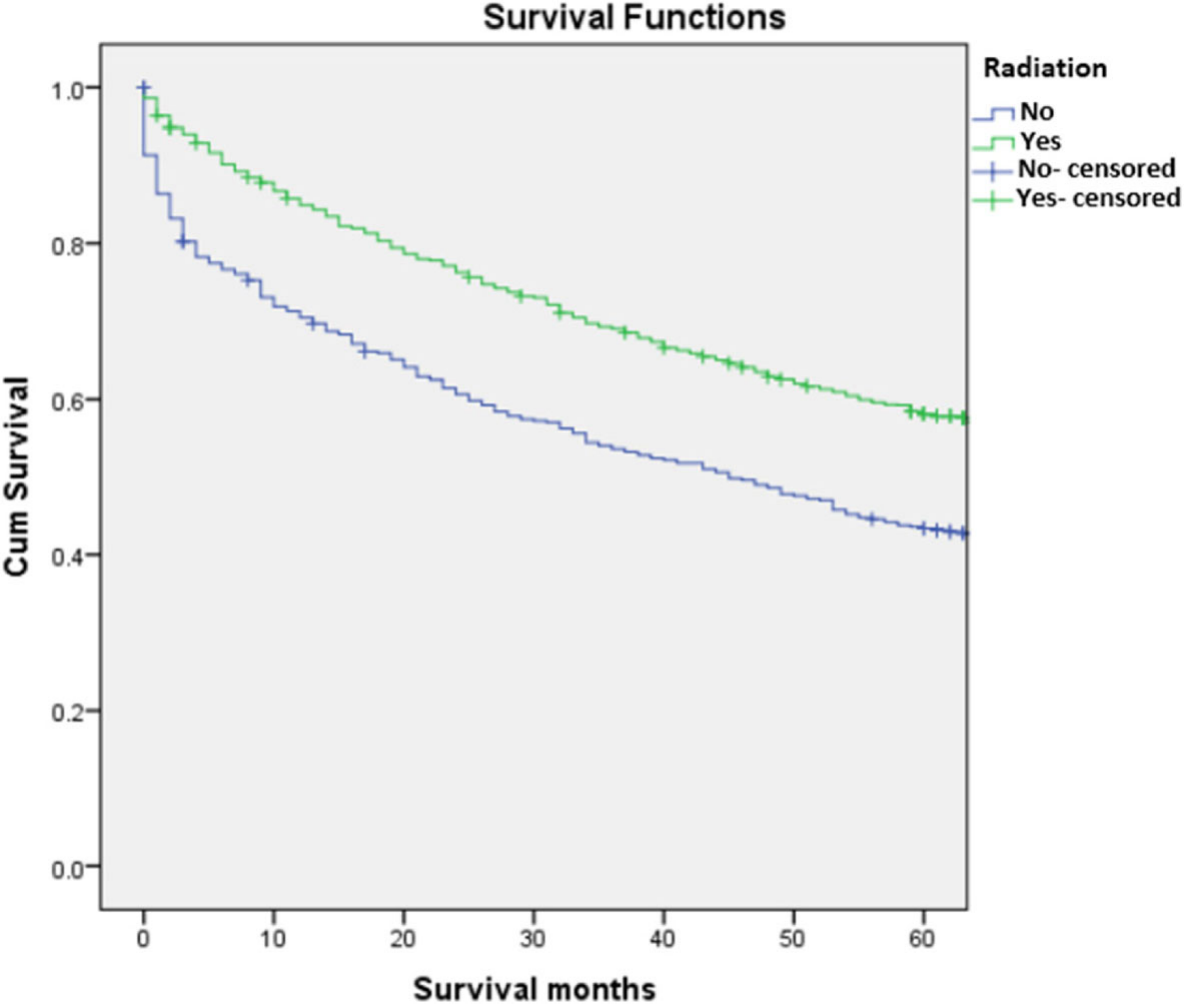
Kaplan-Meier survival curve comparing patients who received RT vs. no RT

**Fig. 3:**
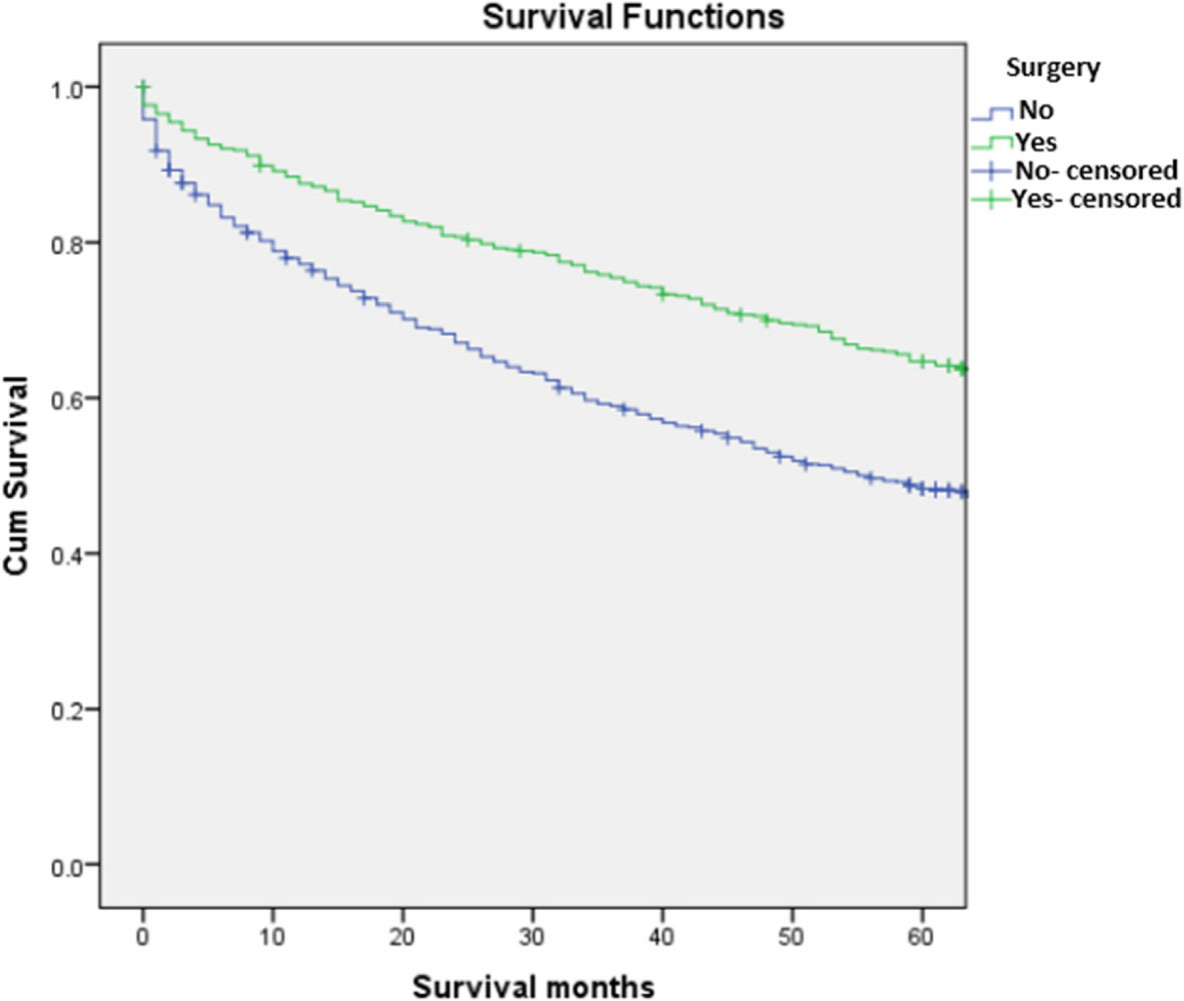
Kaplan-Meier survival curve comparing patients who received surgery vs. those that did not

**Table 6:** Cox proportional hazards model for prognostic factors

Variables	Hazard ratio	95% CI		*P* value
		Lower	Upper	
Age < 60 years	0.380	0.330	0.440	< 0.001
Radiation sequence with surgery	1.226	.966	1.557	.094
Radiation	0.597	0.522	0.684	< 0.001
Surgery	0.746	0.693	0.802	< 0.001

Corrected versions of these figures and tables are included in this Erratum.

Corrected Figure 1

Corrected Figure 2

Corrected Figure 3

Corrected Table 6
