# Supplementary material for: Solitary plasmacytoma: population-based analysis of survival trends and effect of various treatment modalities in the USA
Source: BMC Cancer. 2017 Jan 5;17:13. doi: 10.1186/s12885-016-3015-5 (PMC5216567; doi:10.1186/s12885-016-3015-5)

Additional File 1

Figure S1. Kaplan-Meier survival curve for patients with SP in various age groups.


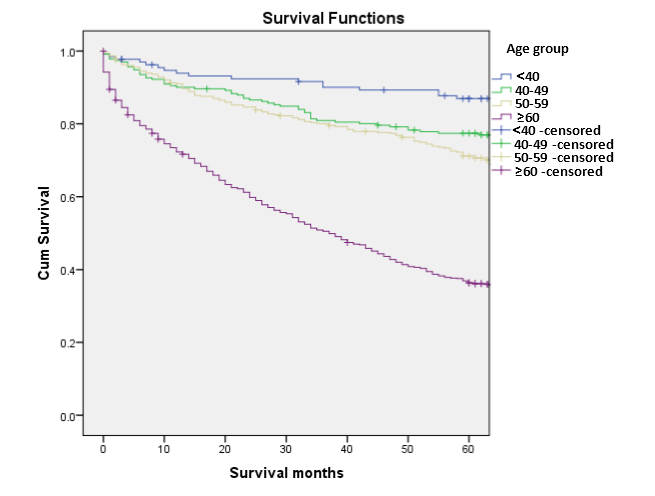


Figure S2. Kaplan-Meier curve for survival rates of males and females.


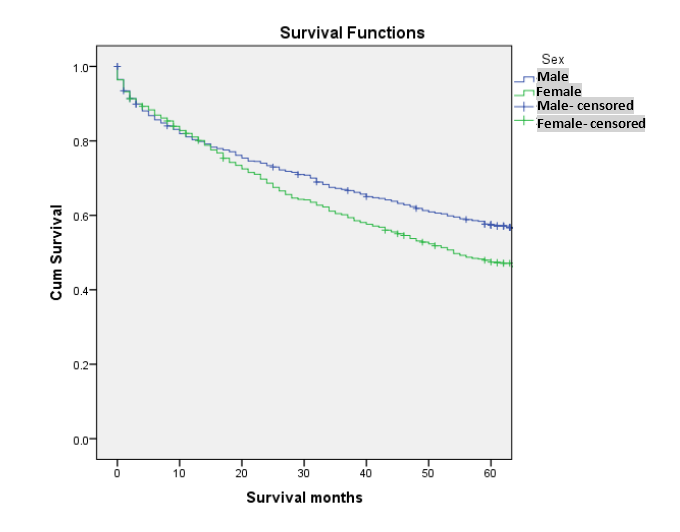


Figure S3: Kaplan-Meier survival curves for patients with plasmacytoma among different races.


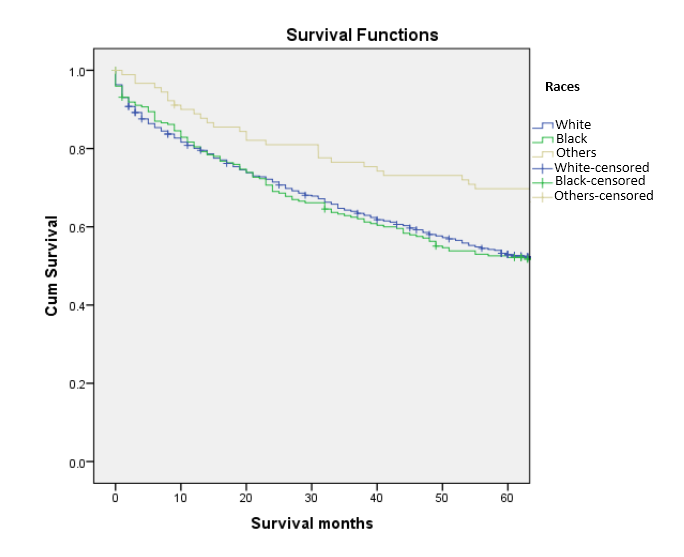

Supplement: Additional file 1: — Kaplan-Meier survival curve for patients with SP in various age groups. (DOCX 140 kb) [file 12885_2016_3015_MOESM1_ESM.docx]
