# Supplementary material for: Solitary plasmacytoma: population-based analysis of survival trends and effect of various treatment modalities in the USA
Source: BMC Cancer. 2017 Jan 5;17:13. doi: 10.1186/s12885-016-3015-5 (PMC5216567; doi:10.1186/s12885-016-3015-5)
Supplement: Additional file 2: — Characteristics comparison between plasmacytoma & myeloma which progressed to myeloma. (DOCX 17 kb) [file 12885_2016_3015_MOESM2_ESM.docx]

Additional File 2

Supplementary Table 1. Characteristics comparison between plasmacytoma & myeloma which progressed to myeloma

| Characteristics | | Plasmacytoma  (N.=553) | | Myeloma  (N.=16852) | |
| --- | --- | --- | --- | --- | --- |
|  |  | N. | % | N. | % |
| Age | >40 | 13 | 2.4% | 201 | 1.2% |
|  | 40-49 | 39 | 7.1% | 1062 | 6.3% |
|  | 50-59 | 102 | 18.4% | 2746 | 16,4% |
|  | ≥60 | 399 | 72.2% | 13242 | 76.1% |
| Sex | Male | 313 | 56.6% | 8707 | 51.7% |
|  | Female | 240 | 43.4% | 8145 | 48.3% |
| Race | White | 449 | 81.2% | 12966 | 76.9% |
|  | Black | 84 | 15.2% | 2977 | 17.7% |
|  | Others | 20 | 3.6% | 909 | 5.4% |
| Exposure To Radiotherapy | Without Radiation | 163 | 29.5% | 12696 | 73.9% |
|  | With Radiation | 390 | 70.5% | 4546 | 26.1% |
| Radiation Sequence | Adjuvant | 95 | 17.2% | 137 | 0.8% |
|  | Neoadjuvant | 2 | 0.4% | 12 | 0.1% |
|  | Neoadjuvant and adjuvant | 1 | 0.2% | 3 | 0% |
| Surgical Intervention | No | 407 | 73.6% | 16732 | 99.3% |
|  | Yes | 146 | 26.4% | 120 | 0.7% |
